# Study on Persulfate Activation and Tetracycline Degradation by Chlorine-Doped Carbon Derived from ZIF-8

**DOI:** 10.3390/molecules31132392

**Published:** 2026-07-07

**Authors:** Wulue Xu, Runhua Chen, Qingwei Wang, Rongkui Su, Yuxia Song, Bo Xiao, Changqing Su

**Affiliations:** 1 School of Ecology and Environment, Central South University of Forestry and Technology, Changsha 410007, China; csuft20231100492@163.com (W.X.); yuxiasong@csuft.edu.cn (Y.S.);; 2 School of Metallurgy and Environment, Central South University, Changsha 410083, China; 3 School of New Energy and Environment, Hunan University of Technology and Business, Changsha 410205, China

**Keywords:** PMS-AOPs, ZIF-8-derived carbon material, sodium chloride, tetracycline

## Abstract

To address the inherent drawbacks of peroxymonosulfate advanced oxidation processes (PMS-AOPs), including the low efficiency of reactive species production, short radical half-lives, and restricted pollutant degradation performance, sodium salt-assisted modification was adopted to fabricate ZIF-8-derived carbon. In this study, sodium salt-assisted modification was adopted to treat ZIF-8, and the chlorine-doped derived carbon materials HNC-T_x_-Cl were prepared for peroxymonosulfate activation and tetracycline degradation in water. Compared with NC-800 fabricated by direct calcination of ZIF-8 at 800 °C, HNC-800-Cl synthesized via NaCl-assisted calcination exhibits more abundant pore structures and richer carbon defects, with a specific surface area of 1115 m^2^/g and a high graphitic defect ratio I_D_/I_G_ of 1.20. Catalytic tests reveal that HNC-800-Cl achieves 93.39% tetracycline removal within 90 min at a catalyst dosage of 0.05 g L^−1^ and PMS concentration of 0.1 mM. The system possesses a strong anti-interference ability toward complex water environments, maintaining a favorable degradation performance in the presence of coexisting anions, natural organic matter and actual water matrices. It also exhibits outstanding cycling stability, retaining a removal rate of 80.34% after five recycling runs. Radical quenching experiments and EPR characterization verify that superoxide radical (·O_2_^−^) is the dominant reactive species during tetracycline degradation. Both the radical and non-radical pathways are clarified to illustrate the mechanisms of PMS activation and pollutant degradation. This work provides a novel catalytic material strategy to overcome the deficiencies of conventional PMS-AOPs, and offers a new perspective for structural regulation and non-metallic doping modification of ZIF-8-derived carbon materials.

## 1. Introduction

With the rapid development of livestock breeding and pharmaceutical industries, China has become the world’s largest consumer of antibiotics [1,2]. As a typical broad-spectrum antibiotic, tetracycline is widely used owing to its low cost and favorable bioavailability [3,4]. However, widespread antibiotic overuse and incomplete wastewater treatment result in persistent tetracycline residues detected in aquatic and soil media [5,6]. Residual tetracycline disturbs the ecological balance, induces antibiotic-resistant bacteria, and endangers human health via food chain accumulation [7,8]. Peroxymonosulfate advanced oxidation processes (PMS-AOPs) generate highly oxidative reactive species after activation, emerging as a mainstream technique for degrading emerging organic water contaminants [9]. Conventional PMS-AOPs suffer from intrinsic drawbacks, including a low efficiency of reactive species generation, short radical half-lives, and weak pollutant mineralization capacity [10]. Common catalysts also suffer from particle agglomeration and metal ion leaching, greatly hindering practical engineering application.

In recent years, carbon-based materials such as biochar, graphene and carbon nanotubes have been widely utilized in PMS advanced oxidation systems owing to their large specific surface area, abundant raw material sources, tunable surface physicochemical properties and favorable anti-interference capability [11,12,13]. Nevertheless, pristine carbon materials lack electron-rich active sites, failing to sustain a stable and efficient catalytic performance during the degradation of emerging contaminants [14]. Metal doping can create local electron-rich domains, introduce structural defects and boost graphitization to elevate catalytic activity, but such modification inevitably causes metal leaching and secondary contamination risks [15,16]. By contrast, non-metallic doping has attracted extensive research attention. It possesses superior charge polarization and ionization properties as well as better thermal stability. Meanwhile, it can precisely regulate the microstructure of carbon materials, optimize specific surface area and electron transfer behavior, facilitate efficient generation of reactive species, and ultimately boost catalytic degradation efficiency [17,18].

Metal–organic frameworks (MOFs) possess distinctive merits including a developed porous structure, high specific surface area, controllable and easily functionalized structure, and convenient composite modification, showing broad application prospects [19,20,21]. As a typical zeolitic imidazolate framework, ZIF-8 can be converted into MOF-derived carbon materials via direct high-temperature calcination under an inert atmosphere, which has been extensively investigated in energy storage, catalysis, gas adsorption and separation [22,23,24]. However, ZIF-8-derived carbon obtained by direct calcination suffers from a disordered internal structure, insufficient catalytic active sites and poor cycling stability, which hardly meets the practical requirements for efficient removal of emerging pollutants in a water environment [25,26]. Accordingly, it is urgent to conduct structural regulation and doping modification on ZIF-8-derived carbon to develop high-performance carbon-based catalysts for efficient elimination of tetracycline pollutants in water.

Aiming at the deficiencies of existing technologies and materials, this study carries out the following research: (1) Chlorine-doped ZIF-8-derived carbon materials are synthesized via a sodium salt-assisted strategy. The physical and chemical structures as well as surface properties are systematically compared between directly calcined ZIF-8 carbon and modified carbon prepared at different calcination temperatures. (2) Taking tetracycline as the target pollutant, the catalytic degradation performance of as-prepared materials for peroxymonosulfate activation is evaluated. (3) The practical application potential of the materials is explored through water matrix interference and cyclic stability tests. (4) Combined with radical quenching experiments and characterization results, the dominant reactive species involved in tetracycline degradation are identified. The degradation pathways and catalytic reaction mechanism are deduced and clarified.

## 2. Results and Discussion

### 2.1. Characterization of Materials

**Figure 1:**
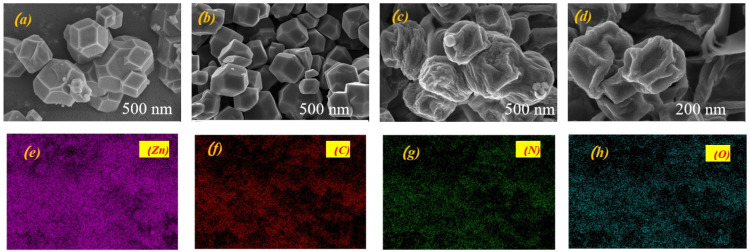
ZIF-8 (**a**), ZIF-8-derived carbon NC-800 obtained by direct calcination at 800 °C (**b**), chlorine-doped carbon HNC-800-Cl synthesized via a sodium salt-assisted strategy at 800 °C under different magnifications (**c**,**d**), and SEM elemental mapping images of HNC-800-Cl (**e**–**h**).

The microscopic morphologies of ZIF-8, NC-800 and HNC-800-Cl were characterized by SEM. As shown in Figure 1a, pure ZIF-8 crystals present a regular dodecahedral structure with distinct edges and corners [27]. Figure 1b shows NC-800 obtained via direct high-temperature pyrolysis of ZIF-8. It retains a dodecahedral outline but exhibits irregular particle sizes and smoother surfaces with deteriorated structural integrity. Figure 1c,d show SEM images of HNC-800-Cl at different magnifications. After sodium salt-assisted modification, the intrinsic dodecahedral skeleton of ZIF-8 is well preserved. Numerous folds and rough structures emerge on the material surface under high magnification. The reconstructed microstructure contributes to abundant pores and an enlarged specific surface area, providing sufficient active sites for pollutant adsorption and catalytic reaction, thereby facilitating tetracycline degradation in water [28,29]. Figure 1e–h illustrate the elemental mapping of HNC-800-Cl. Zn, C, N and O elements are uniformly distributed over the material surface with favorable dispersion. Combined morphological and elemental distribution results confirm the successful synthesis of the chlorine-doped ZIF-8-derived carbon HNC-800-Cl.

**Figure 2:**
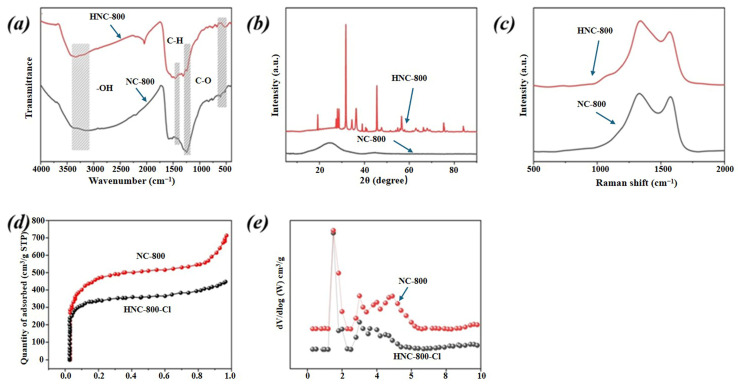
FTIR spectra (**a**), XRD patterns (**b**), Raman spectra (**c**), N_2_ adsorption–desorption isotherms (**d**) and the pore size distribution (**e**) of NC-800 and HNC-800-Cl.

Fourier transform infrared spectroscopy was employed to compare the surface functional groups and structural differences between NC-800 and HNC-800-Cl. As illustrated in Figure 2a, both samples exhibit obvious stretching vibration absorption peaks near 500 cm^−1^, assigned to the characteristic vibration of Zn–O-C bonds, proving the existence of zinc oxide-related structures on material surfaces [30]. The absorption peaks at 1100 cm^−1^ and 1500 cm^−1^ correspond to C–O and C–H bonds, respectively, revealing abundant organic functional groups on both carbon materials [31]. The broad peak ranging from 3000 to 3500 cm^−1^ belongs to the stretching vibration of surface hydroxyl groups (-OH) [32]. HNC-800-Cl exhibits significantly stronger absorbance within this range than NC-800, confirming enhanced surface hydrophilicity after NaCl-assisted thermal treatment. In general, the two samples share similar peak shapes and characteristic peak positions, indicating chlorine doping assisted by sodium salt does not destroy the basic functional group skeleton of the original materials.

X-ray diffraction patterns were used to analyze the crystal phase composition. Figure S9 displays the distinct characteristic diffraction peaks of ZIF-8 at 2θ of 10.4°, 12.8° and 18.1°, corresponding to the (002), (112) and (222) crystal planes, respectively, which matches well with the standard crystal phases reported in the literature and verifies successful synthesis of the ZIF-8 precursor [33,34]. According to Figure 2b, the residual characteristic peaks of ZIF-8 and typical peaks of NaCl are simultaneously detected in HNC-800-Cl, confirming successful chlorine doping into the ZIF-8-derived carbon skeleton [35]. No diffraction signals of elemental zinc or zinc oxides are observed, implying no precipitation of zinc crystalline phases after calcination modification, which effectively avoids secondary pollution caused by metal leaching [36].

The Raman spectra were measured to explore the structural defects and graphitization degree of the carbon materials. As shown in Figure 2c, both NC-800 and HNC-800-Cl present typical D-band and G-band peaks around 1350 cm^−1^ and 1575 cm^−1^. The D-band reflects structural defects and disordered carbon, while the G-band represents the sp^2^ conjugated graphitic carbon skeleton. The calculated I_D_/I_G_ ratio was 1.05 for NC-800 and increased to 1.20 for HNC-800-Cl. The Raman profiles of the materials pyrolyzed at varied temperatures are provided in Figure S6. The results prove that sodium chloride-assisted modification can substantially increase surface defect sites. Rich structural defects serve as efficient catalytic active centers, facilitating peroxymonosulfate activation and enhancing tetracycline degradation performance [37].

**Figure 3:**
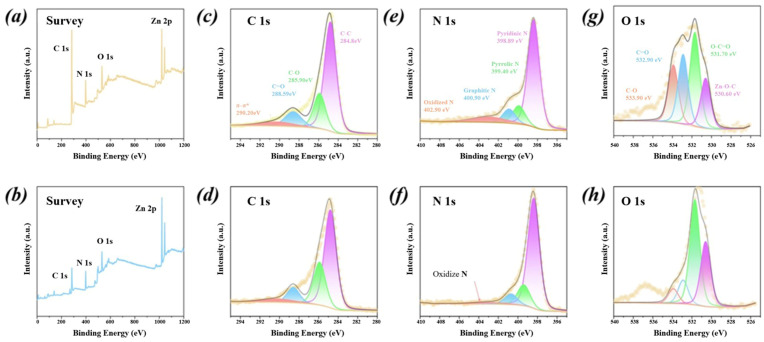
Survey XPS spectra of NC-800 and HNC-800-Cl (**a**,**b**); high-resolution XPS spectra of C 1s (**c**,**d**), N 1s (**e**,**f**) and O 1s (**g**,**h**).

X-ray photoelectron spectroscopy was applied to analyze the surface elemental composition, chemical states and bonding configurations of NC-800 and HNC-800-Cl. The full survey spectra in Figure 3a,b display strong characteristic peaks at 284 eV, 399 eV and 533 eV, attributed to C 1s, N 1s and O 1s, consistent with the SEM elemental mapping results. The relative elemental contents are summarized in Table S1. Four fitted peaks are obtained from the deconvoluted C 1s spectra (Figure 3c,d), corresponding to a C–C bond at 284.80 eV, C–O bond at 285.90 eV, C=O bond at 288.59 eV and π–π* conjugated carbon structure at 290.20 eV [38]. The proportions of various carbon configurations are listed in Table S2. The N 1s spectra (Figure 3e,f) are divided into four nitrogen species: pyridinic N at 398.89 eV, pyrrolic N at 399.40 eV, graphitic N at 400.90 eV and oxidized N at 402.90 eV [39]. Their distribution ratios are presented in Table S3. Pyridinic N and graphitic N act as high-efficiency active sites for peroxymonosulfate activation. Four characteristic peaks are also fitted from the O 1s spectra (Figure 3g,h), assigned to a Zn-O-C bond at 530.60 eV, O-C=O bond at 531.70 eV, C=O bond at 532.90 eV and C-O bond at 533.90 eV [40]. The contents of oxygen-containing functional groups are summarized in Table S4. Abundant nitrogen and oxygen functional groups provide sufficient active sites for catalytic reactions.

N_2_ adsorption–desorption isotherms were tested to characterize the pore structure and specific surface area. As depicted in Figure 3e, rapid adsorption capacity growth at low pressure (P/P_0_ < 0.2) manifests abundant micropores inside materials. The gentle adsorption curve and obvious hysteresis loop within the P/P_0_ of 0.5~1.0 indicate the coexistence of mesopores. The isotherm conforms to type IV standard characteristics. The N_2_ adsorption–desorption curves and pore size distribution of samples prepared at different temperatures are shown in Figures S7 and S8. Combined with Figure 3f and the pore parameters in Table S9, NC-800 exhibited a specific surface area of 976 m^2^ g^−1^ and micropore volume of 0.32 cm^3^ g^−1^. After NaCl modification, HNC-800-Cl delivered a larger specific surface area (1115 m^2^ g^−1^) and higher micropore volume (0.56 cm^3^ g^−1^). It is demonstrated that sodium chloride can effectively regulate the pore structure of ZIF-8-derived carbon, enlarge the specific surface area and micropore volume, and supply adequate interfacial space and active sites for pollutant adsorption, mass transfer and catalytic degradation.

### 2.2. Catalytic Performance

**Figure 4:**
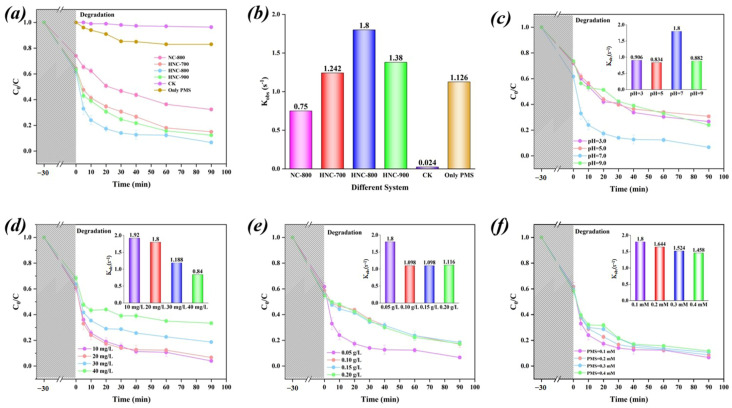
Effects of different materials on the Fenton-like degradation of tetracycline (**a**,**b**); influences of solution pH (**c**), tetracycline concentration (**d**), catalyst dosage (**e**) and PMS concentration (**f**) on tetracycline degradation by HNC-800-Cl.

The Fenton-like catalytic performance of HNC-800-Cl was evaluated via tetracycline degradation. The adsorption kinetic test curve is shown in Figure S11. This curve clearly demonstrates that the adsorption–desorption saturation state is completely reached at 30 min. After reaching adsorption–desorption equilibrium within 30 min, peroxymonosulfate (PMS) was added to initiate the Fenton-like reaction. Figure 4a,b show the optimization of the calcination temperature by comparing the degradation capacity of HNC-T_x_-Cl synthesized at different temperatures.

The ZIF-8@NaCl composites obtained via sodium salt assistance exhibited markedly enhanced tetracycline removal efficiency compared with directly calcined carbon materials. The optimal performance was achieved by HNC-800-Cl calcined at 800 °C, with a removal efficiency of 93.39% within 90 min. The degradation rate constant increased from 0.7500 s^−1^ to 1.8000 s^−1^. No further performance improvement was observed for HNC-900-Cl. Since NaCl melts at 800 °C, it volatilized at 900 °C and exposed carbon particles to extreme heat, destroying the intrinsic structure of the ZIF-8-derived carbon. HNC-700-Cl delivered inferior degradation efficiency, because the protective effect of NaCl hinders complete graphitization of the internal ZIF-8 crystals at a relatively low temperature [27].

The solution pH was adjusted with 1 mol/L HCl and 1 mol/L NaOH to explore pH influence. Within the pH range of 3–9, the maximum degradation efficiency was obtained at neutral pH 7. The removal rates dropped to 73.05%, 69.26% and 76.20% at pHs of 3, 5 and 9, respectively. At pH < 3, tetracycline primarily exists as positively charged TC^+^, and electrostatic repulsion between the positively charged catalyst surface and TC^+^ suppressed adsorption and catalytic reactions. The neutral condition favors the neutral TC° form, dominated by the van der Waals force and hydrogen bonding, hence the optimal degradation behavior. Under alkaline conditions, tetracycline presents negative forms of TC^−^ and TC^2−^. Electrostatic repulsion occurs among the negatively charged catalyst, tetracycline and PMS, restraining PMS activation and pollutant elimination [41,42].

The effect of the initial tetracycline concentration was investigated in Figure 4d. The highest removal efficiency of 95.74% and rate constant of 1.9200 s^−1^ were achieved at 10 mg/L. As the pollutant concentration rose to 40 mg/L, the removal efficiency declined to 66.57% and the rate constant decreased to 0.8400 s^−1^. Elevated contaminant concentration causes rapid occupation and fierce competition over limited active sites. High-concentration tetracycline also inhibits catalyst activity and impedes the degradation reaction [43].

Figure 4e,f illustrate the effects of the catalyst dosage and PMS concentration. The degradation efficiency did not rise continuously with the increasing catalyst or oxidant dosage, showing a consistent declining trend. On one hand, insufficient PMS cannot match the excessive catalyst quantity. On the other hand, the superfluous oxidant triggers self-quenching reactions among reactive oxygen species such as sulfate radicals and peroxymonosulfate ions, reducing the number of valid reactive species for tetracycline degradation [44,45].

### 2.3. Application Potential

To evaluate the practical application potential and cyclic stability of the HNC-800-Cl catalytic system for tetracycline degradation, the effects of common coexisting anions, humic acid, and actual water matrices were investigated. Repeated cycling tests were also conducted to verify catalyst reusability.

**Figure 5:**
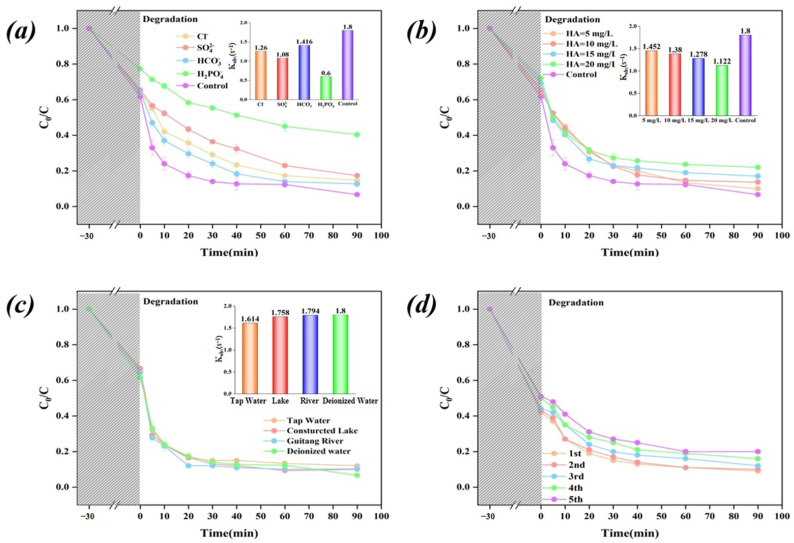
Effects of coexisting inorganic ions (**a**), humic acid (**b**) and different water matrices (**c**) on tetracycline degradation by the HNC-800-Cl Fenton-like system; the cyclic stability test of HNC-800-Cl (**d**).

The influences of the typical coexisting anions Cl^−^, SO_4_^2−^, HCO_3_^−^ and H_2_PO_4_^−^ were firstly analyzed. As shown in Figure 5a, all anions imposed inhibitory effects to varying degrees. H_2_PO_4_^−^ exhibited the strongest suppression, lowering the tetracycline removal efficiency to 59.65%. In comparison, Cl^−^, SO_4_^2−^ and HCO_3_^−^ showed mild inhibition, with removal efficiencies staying above 80% at 85.35%, 82.56% and 87.23%, respectively.

The humic acid concentration exerted negative impacts on catalytic performance. According to Figure 5b, the tetracycline removal efficiency and reaction rate constant gradually decreased with an increasing humic acid concentration. The original removal efficiency reached 93.59% with a rate constant of 1.800 s^−1^ in the absence of humic acid. When the humic acid concentration increased to 20 mg/L, the removal efficiency dropped to 77.97% and the rate constant declined to 1.002 s^−1^. High-concentration natural organic matter weakens the degradation efficiency via competing for active sites and quenching reactive species [46].

Tap water, river water and lake water were adopted as practical water matrices. Figure 5c presents the removal efficiencies of 88.22%, 90.04% and 89.51%, separately. The slight discrepancy confirms the favorable adaptability and anti-interference capacity of the catalytic system towards complex natural water environments.

Five consecutive cycling experiments were carried out to assess reusability and structural stability. As displayed in Figure 5d, the removal efficiency slightly decreased from 90.68% to 80.34% after five runs and still remained at a relatively high level. The chlorine-doped carbon catalyst possesses outstanding cyclic stability and structural durability, satisfying the demands for continuous pollutant treatment in actual water bodies.

In conclusion, the HNC-800-Cl catalytic system delivers strong resistance against anions and natural organic matter, a favorable adaptability to actual water, and excellent reusability. It holds promising engineering application prospects for tetracycline contamination remediation in a water environment. Zn^2+^ leaching tests under various pH conditions are shown in Table S11. The extremely low Zn dissolution level indicates that HNC-800-Cl possesses excellent structural durability, and the risk of secondary metal contamination is low.

### 2.4. Mechanism Analysis

**Figure 6:**
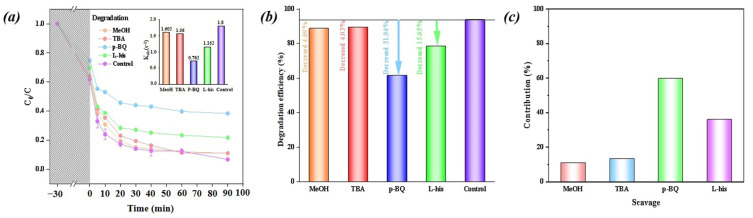
Effects of different reactive species quenchers on tetracycline degradation (**a**), the corresponding removal efficiency (**b**), and the relative contribution of reactive oxygen species (**c**).

**Figure 7:**
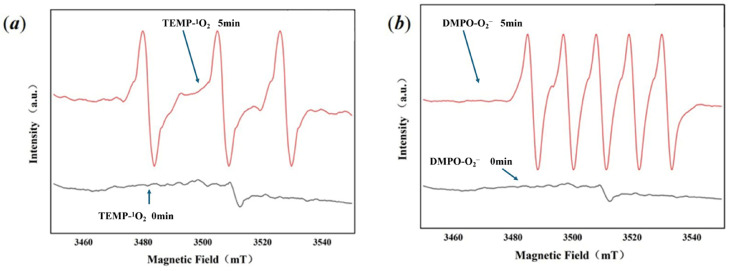
EPR spectra of ^1^O_2_ and ·O_2_^-^ at different reaction times (**a**,**b**).

Radical selective quenching experiments were performed to clarify the contribution mechanism of various reactive species during tetracycline degradation in the HNC-800-Cl-mediated Fenton-like system. As shown in Figure 6a–c, MeOH, TBA, p-BQ and L-his were adopted as specific quenchers. The corresponding tetracycline removal efficiencies were 88.69%, 89.35%, 61.44% and 78.33%, with apparent reaction rate constants of 1.6020 s^−1^, 1.5600 s^−1^, 0.7260 s^−1^ and 1.1520 s^−1^, respectively. Quantitative calculations indicated that the relative contributions of ·OH, ·SO_4_^−^, ^1^O_2_ and ·O_2_^−^ were 11.00%, 13.33%, 59.67% and 36.00%, respectively. The sum of these values surpassed 100% owing to complex interconversion pathways among diverse reactive species. Mutual conversion and coupled generation exist among different reactive species. Besides quenching targeted radicals, quenchers interfere with chain radical reactions and alter the generation and transformation pathways, resulting in a sum of contribution rates over 100% [47]. Quenching results indicated ·O_2_^−^ acted as the dominant reactive species, followed by ^1^O_2_. EPR characterization was conducted for qualitative verification. The typical 1:1:1 signal peaks corresponding to TEMP-^1^O_2_ and DMPO-O_2_^−^ adducts were observed in Figure 7a,b. The signal intensity of DMPO-·O_2_^−^ was obviously higher, confirming ·O_2_^−^ as the predominant reactive species, which was consistent with the quenching experimental results. Based on the above findings, the degradation mechanism involving radical and non-radical pathways was illustrated.

Radical pathway PMS was activated by HNC-800-Cl and decomposed into electrons, hydrogen ions and ·SO_5_^−^ (Equation (1)). Electrons reacted with dissolved oxygen and HSO_5_^−^ to produce ·OH, ·SO_4_^−^ and ·O_2_^−^ simultaneously (Equations (2)–(4)). HSO_5_^−^ reacted with water to form H_2_O_2_ and HSO_4_^−^. The yielded H_2_O_2_ further reacted with ·OH to generate ·HO_2_, which self-decomposed and continuously supplemented ·O_2_^−^ (Equations (5)–(7)).

Non-radical pathway ^1^O_2_ was directly produced via interaction between ·OH and H_2_O_2_ (Equation (8)). ·SO_4_^−^ participated in a chain reaction with HSO_5_^−^ to generate SO_4_^2−^, ·SO_5_^−^ and H^+^, and the newly formed ·SO_5_^−^ reacted with water to yield ^1^O_2_ (Equations (9) and (10)). In addition, the coupling reaction between ·O_2_^−^ and ·OH also facilitated the formation of ^1^O_2_ (Equation (11)).

Reactive species from dual pathways attacked tetracycline molecules, destroying conjugated structures and functional skeletons. Macromolecular tetracycline was gradually degraded into small intermediates and finally mineralized into carbon dioxide and water to achieve thorough pollutant elimination (Equation (12)).

Catalyst + PMS → e^−^ + H^+^ + SO_5_^−^ (1)

e^−^ + O_2_ → ·O_2_^-^ (2)

e^−^ + HSO_5_^−^ → ·OH + SO_4_^2−^ (3)

e^−^ + HSO_5_^−^ → ·SO_4_^−^ + HO^−^ (4)

HSO_5_^−^ + H_2_O → H_2_O_2_ + HSO_4_^−^ (5)

H^2^O^2^ + ·OH → ·HO_2_^−^ + H_2_O (6)

·HO_2_^−^ → ·O_2_^−^ + H_2_O (7)

2·OH + H_2_O_2_ → ^1^O_2_ + H_2_O (8)

HSO_5_^−^ + ·SO_4_^−^ → SO_4_^2−^ + ·SO_5_^−^ + H^+^ (9)

2·SO_5_^−^ + H_2_O → ^1^O_2_ + 2SO_4_^2−^ + 2H^+^ (10)

O_2_·^−^ + ·OH + H^+^ → ^1^O_2_ + H_2_O (11)

·OH/·SO_4_^−^/·O_2_^−^/^1^O_2_ + TC → intermediate → CO_2_ + H_2_O (12)

### 2.5. The Possible Degradation Pathway

**Figure 8:**
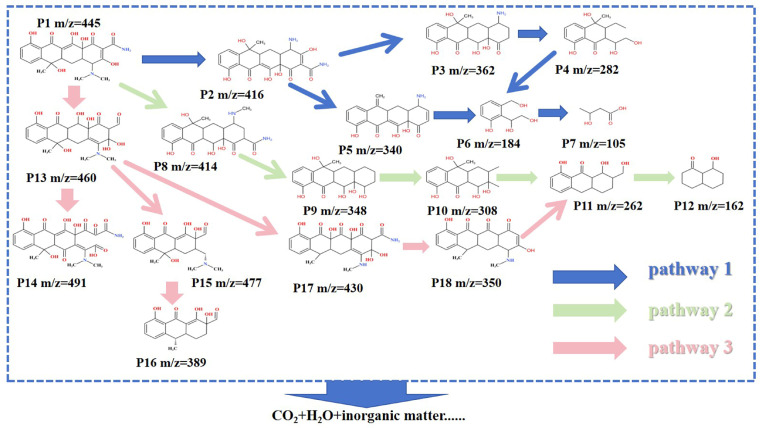
Possible degradation pathways of tetracycline in the HNC-800-Cl/PMS system.

Liquid chromatography–mass spectrometry (LC-MS) was applied to identify intermediate products so as to clarify the degradation pathway of tetracycline in the HNC-800-Cl/PMS system. Water samples collected at 0 min, 10 min and 30 min were analyzed via full mass spectrum scanning. A total of 18 potential intermediates were identified according to mass-to-charge ratios, and the detailed substance information and corresponding *m*/*z* values are listed in Table S10. The structures of TC and radicals are shown in Figure S10a, and the potential energy surface for the reaction between TC and the radicals is shown in Figure S10b. Three major degradation pathways were summarized on the basis of the mass spectral data and literature references, as illustrated in Figure 7. Pathway 1: The N–C bond cleavage and demethylation firstly occur on tetracycline molecules to form intermediate P2 (*m*/*z* = 416). P2 degrades through two sub-routes. In the first route, reactive species preferentially attack aromatic rings, carbon–carbon double bonds and phenolic hydroxyl groups to produce P3 (*m*/*z* = 362), which is further oxidized into P4 (*m*/*z* = 282). Subsequent ring opening, deamination, hydroxylation and deep oxidation reactions successively yield P6 (*m*/*z* = 184) and P7 (*m*/*z* = 105). In the second route, P2 transforms into P5 (*m*/*z* = 340) via dehydration, demethylation and deamination. The cyclic skeleton of P5 breaks under radical oxidation to generate P6 (*m*/*z* = 184), which is eventually degraded into small-molecule product P7 (*m*/*z* = 105). Pathway 2: Tetracycline is modified by demethylation and hydroxylation to generate P8 (*m*/*z* = 414). Under radical oxidation, P8 undergoes demethylation, deamidation and hydrogenation to form P9 (*m*/*z* = 348) and P10 (*m*/*z* = 308). P10 experiences continuous demethylation, dehydroxylation, decarboxylation and ring opening to obtain P11 (*m*/*z* = 262). Further dehydroxylation, decarboxylation and ring cleavage convert P11 into P12 (*m*/*z* = 162). Pathway 3: Carbon ring oxidation of tetracycline produces P13 (*m*/*z* = 460), which is further oxidized to P14 (*m*/*z* = 491). P13 follows two degradation branches. Deep oxidation of P13 generates P15 (*m*/*z* = 477), and subsequent oxidative dehydrogenation and successive demethylation produce P16 (*m*/*z* = 389). Alternatively, partial demethylation and dehydroxylation of P13 forms P17 (*m*/*z* = 430). Complete dimethylamine removal and dehydroxylation of P17 yields P11 (*m*/*z* = 262) and P18 (*m*/*z* = 262). The resulting intermediates undergo sustained dehydroxylation, decarboxylation and ring cracking, and finally convert into P12 (*m*/*z* = 162). All intermediates generated from the three pathways can be thoroughly degraded and mineralized under continuous radical oxidation, ultimately turning into carbon dioxide, water and non-toxic small molecules, thus achieving the harmless removal of tetracycline. The comparison of the toxicity assessment has been listed in Table S11. These results demonstrate that the HNC-800-Cl/PMS system exhibits outstanding environmental safety and practical application potential. The complete transformation sequence of tetracycline and corresponding intermediate products under HNC-800-Cl/PMS oxidation are visually displayed in Figure 8.

## 3. Materials and Methods

### 3.1. Materials

Zinc nitrate hexahydrate (Zn(NO_3_)_2_·6H_2_O) was purchased from Aladdin Biochemical Technology Co., Ltd. (Shanghai, China). Tetracycline hydrochloride (C_22_H_24_N_2_O_8_.HCl), potassium peroxymonosulfate (KHSO_5_, PMS), 2-methylimidazole (C_4_H_6_N_2_) and methanol (CH_3_O) were obtained from Macklin Biochemical Technology Co., Ltd. (Shanghai, China). Sodium chloride (NaCl) and sodium hydroxide (NaOH) were supplied by Sinopharm Chemical Reagent Co., Ltd. (Shanghai, China). Hydrochloric acid (HCl) was purchased from Kelong Chemical Co., Ltd. (Chengdu, China). All other reagents were of analytical grade, and ultrapure water was applied throughout the experiments.

### 3.2. Preparation of Materials

#### 3.2.1. Preparation of ZIF-8

Zinc nitrate hexahydrate and 2-methylimidazole were weighed at a molar ratio of 1:8 and dissolved in 50 mL methanol, respectively. The mixtures were ultrasonically treated and magnetically stirred until fully dissolved to obtain two precursor solutions, A and B. Solution B was slowly dropped into solution A, followed by continuous stirring at room temperature for 24 h. The resultant product was centrifugally washed three times with methanol and vacuum freeze-dried to acquire ZIF-8 precursor powder.

#### 3.2.2. Preparation of HNC-T_x_-Cl

Exactly 100 mg of as-synthesized ZIF-8 powder (Section 3.2.1) was dispersed in 10 mL NaCl aqueous solution, followed by vigorous stirring at room temperature for 24 h. The solvent was evaporated at a constant temperature of 80 °C to obtain white crystalline precursors. The samples were placed in a tube furnace and calcined at 700 °C, 800 °C and 900 °C for 2 h under a nitrogen atmosphere, denoted as HNC-700-Cl, HNC-800-Cl and HNC-900-Cl, respectively. After natural cooling to room temperature, the samples were washed with ultrapure water and 1 mol/L hydrochloric acid to remove templates, and dried for subsequent use. Meanwhile, equal amounts of ZIF-8 powder were directly calcined at 800 °C for 2 h under a nitrogen atmosphere to prepare the undoped ZIF-8-derived carbon material named NC-800.

### 3.3. Degradation Experiment

Degradation experiments were carried out in 250 mL conical flasks. A total of 100 mL tetracycline (TC) solution was transferred into the flask, followed by the addition of a certain amount of catalyst under continuous stirring. The mixture was kept in the dark for 30 min to achieve adsorption–desorption equilibrium between the catalyst and TC. Peroxymonosulfate (PMS) was then added to initiate the catalytic degradation reaction. Then, 2 mL aliquots were withdrawn at predetermined time points and filtered via 0.45 μm water-phase filter membranes. An equal volume of sodium thiosulfate solution was instantly added to terminate oxidation reactions. The residual tetracycline concentration in filtrate was determined by Agilent (Santa Clara, CA, USA) 1260 Infinity high performance liquid chromatography.

### 3.4. Characterization

The microscopic morphology of samples was observed via a Zeiss Sigma (Oberkochen, Germany) 300 scanning electron microscope (SEM), and the micro-area elemental composition was qualitatively analyzed by a supporting EDS spectrometer. The specific surface area and pore structure were measured using a Micromeritics (Norcross, GA, USA) ASAP2460 BET analyzer. The crystal phase structure was characterized by an Oxford Xplore X-ray diffractometer (XRD). The types and variation of surface functional groups were analyzed with a Thermo Fisher Nicolet (Waltham, MA, USA) 6700 Fourier transform infrared spectrometer (FTIR). The defect degree and graphitization property of carbon materials were detected by a HORIBA (Singapore) HRS 600 Raman spectrometer. A Thermo Fisher ESCALAB Xi+ X-ray photoelectron spectrometer (XPS) was adopted to investigate the surface elemental composition, chemical valence and bonding state variations.

## 4. Conclusions

In this study, sodium chloride was adopted as a salt template to modify ZIF-8, and a chlorine-doped ZIF-8-derived carbon catalyst was successfully synthesized. The material was applied to activate peroxymonosulfate for efficient tetracycline degradation in water. The constructed HNC-800-Cl/PMS system exhibited prominent catalytic performance, achieving 93.39% tetracycline removal within 90 min. The system possessed strong tolerance against coexisting ions, natural organic matter and actual water matrices, along with satisfactory recyclability, showing great potential for practical engineering application. Comparison of the Cl-doped ZIF-8 derived carbon (HNC-800-Cl) and other materials are summarized and listed in Table S12. As shown in Table S12, sodium salt-assisted Cl doping optimizes the pore structure and enriches surface defects, endowing the catalyst with superior catalytic activity and anti-interference capacity. Radical quenching tests and EPR characterization verified the superoxide radical (·O_2_^−^) served as the dominant reactive species, followed by singlet oxygen (·O_2_^−^). The favorable reactive species generation capacity originated from the strong peroxymonosulfate activation ability of the chlorine-doped carbon material. Combined with HPLC-MS intermediate identification results, multiple degradation pathways were elaborated. Under the continuous attack of reactive species, tetracycline underwent bond cleavage, functional group elimination, benzene ring opening and deep oxidation, gradually degraded into small molecular intermediates and finally mineralized completely. This work provides a feasible strategy for microstructure regulation and non-metallic chlorine doping modification of ZIF-8-derived carbon. It also offers novel insights and a theoretical reference for designing high-performance environmental catalysts and establishing persulfate advanced oxidation systems to eliminate emerging antibiotic contaminants in water.

## Data Availability

The original contributions presented in this study are included in the article/Supplementary Material. Further inquiries can be directed to the corresponding author.

## Supplementary Materials

The following supporting information can be downloaded at https://www.mdpi.com/article/10.3390/molecules31132392/s1: Text S1: Strategy for preparing derived carbon materials from ZIF-8 via the salt-assisted method. Text S2: Analysis method. Figure S1: The schematic chart of HNC-Tx-Cl. Figure S2: Full XPS survey spectrum of HNC-800-Cl after tetracycline degradation. Figure S3: High-resolution C 1s XPS spectrum of HNC-800-Cl after tetracycline degradation. Figure S4: High-resolution N 1s XPS spectrum of HNC-800-Cl after tetracycline degradation. Figure S5: High-resolution O 1s XPS spectrum of HNC-800-Cl after tetracycline degradation. Figure S6: Raman spectra of HNC-700-Cl and HNC-900-Cl. Figure S7: N_2_ adsorption–desorption isotherms of HNC-700-Cl and HNC-900-Cl. Figure S8: Pore size distribution of HNC-700-Cl and HNC-900-Cl. Figure S9: XRD pattern of ZIF-8. Figure S10: (**a**) The structures of TC and radicals; (**b**) the potential energy surface for the reaction between TC and radicals. Figure S11: Adsorption kinetic test curve. Table S1: Elemental proportions from XPS of NC and HNC-800-Cl. Table S2: Relative contents of various carbon species in C 1s XPS spectra of NC and HNC-800-Cl. Table S3: Relative proportions of different nitrogen species in N 1s XPS spectra of NC and HNC-800-Cl. Table S4: Relative contents of various oxygen species in O 1s XPS spectra of NC and HNC-800-Cl. Table S5: Elemental composition of HNC-800-Cl after degradation via XPS analysis. Table S6: Relative contents of carbon species in C 1s XPS spectra of HNC-800-Cl after degradation. Table S7: Relative contents of nitrogen species in N 1s XPS spectra of HNC-800-Cl after degradation. Table S8: Relative contents of oxygen species in O 1s XPS spectra of HNC-800-Cl after degradation. Table S9: Pore structure parameters of NC-800 and HNC-T_x_-Cl. Table S10: Main intermediates during tetracycline degradation in the HNC-800-Cl/PMS system. Table S11: Comparison of toxicity assessment. Table S12: Zn leaching concentrations. Table S13: Comparison of Cl-doped ZIF-8-derived carbon (HNC-800-Cl) and other materials. Refs. [45,48,49,50,51] are cited in the Supplementary Materials.
