# Supplementary material for: Study on Persulfate Activation and Tetracycline Degradation by Chlorine-Doped Carbon Derived from ZIF-8
Source: Molecules. 2026 Jul 7;31(13):2392. doi: 10.3390/molecules31132392 (PMC13363552; doi:10.3390/molecules31132392)
Supplement: Supplementary file 1 [file molecules-31-02392-s001.zip › molecules-4371563-supplementary.pdf]

Supporting Information

Study on Persulfate Activation and Tetracycline Degradation by Chlorine-Doped Carbon Derived from ZIF-8

Wulue Xu [1], Runhua Chen [1,*], Qingwei Wang [2], Rongkui Su [1], Yuxia Song [1], Bo Xiao [1] and Changqing Su [3]

[1] School of Ecology and Environment, Central South University of Forestry and Technology, Changsha 410007, China; csuft20231100492@163.com (W.X.); yuxiasong@csuft.edu.cn (Y.S.)

[2] School of Metallurgy and Environment, Central South University, Changsha 410083, China

[3] School of New Energy and Environment, Hunan University of Technology and Business, Changsha 410205, China

* Correspondence: chen12@csuft.edu.cn; Tel.: +86-15116121051; Fax: +86-731-81847773

This file include

Text S1 Strategy for preparing derived carbon materials from ZIF-8 via the salt-assisted method

Text S2 Analysis method

Figure S1 The schematic chart of HNC-T_x-Cl

Figure S2 Full XPS survey spectrum of HNC-800-Cl after tetracycline degradation

Figure S3 High-resolution C 1s XPS spectrum of HNC-800-Cl after tetracycline degradation

Figure S4 High-resolution N 1s XPS spectrum of HNC-800-Cl after tetracycline degradation

Figure S5 High-resolution O 1s XPS spectrum of HNC-800-Cl after tetracycline degradation

Figure S6 Raman spectra of HNC-700-Cl and HNC-900-Cl

Figure S7 N_2 adsorption–desorption isotherms of HNC-700-Cl and HNC-900-Cl

Figure S8 Pore size distribution of HNC-700-Cl and HNC-900-Cl

Figure S9 XRD pattern of ZIF-8

Figure S10 (a) The structures of TC and radicals;(b) the potential energy surface for the reaction between TC and radicals

Figure S11 Adsorption kinetic test curve

Table S1 Elemental proportions from XPS of NC and HNC-800-Cl

Table S2 Relative contents of various carbon species in C 1s XPS spectra of NC and HNC-800-Cl

Table S3 Relative proportions of different nitrogen species in N 1s XPS spectra of NC and HNC-800-Cl

Table S4 Relative contents of various oxygen species in O 1s XPS spectra of NC and HNC-800-Cl

Table S5 Elemental composition of HNC-800-Cl after degradation via XPS analysis

Table S6 Relative contents of carbon species in C 1s XPS spectra of HNC-800-Cl after degradation

Table S7 Relative contents of nitrogen species in N 1s XPS spectra of HNC-800-Cl after degradation

Table S8 Relative contents of oxygen species in O 1s XPS spectra of HNC-800-Cl after degradation

Table S9 Pore structure parameters of NC-800 and HNC-T_x-Cl

Table S10 Main intermediates during tetracycline degradation in the HNC-800-Cl/PMS system

Table S11 Comparison of toxicity assessment

Table S12 Zn leaching concentrations

Table S13 Comparison of Cl-doped ZIF-8-derived carbon (HNC-800-Cl) and other materials

Text S1 Strategy for Preparing Derived Carbon Materials from ZIF-8 via Salt-assisted Method

During high-temperature calcination of MOF materials, mutual restriction exists between organic ligands and metal oxides. Organic ligands regulate the nucleation and growth of metal oxides, while the evolution of metal oxides reversely affects the condensation reaction of organic ligands. Their synergistic effect jointly determines the micromorphology and skeleton structure of calcined products. Reasonably coordinating their interaction is of great significance for precise structure regulation of MOF-derived carbon materials.

Salt template method can effectively achieve the above regulation. Salt recrystallization encapsulates MOF precursors in confined spaces formed by salts to ensure uniform heating. Most salts possess high melting points and serve as structural supports at high temperatures, preventing structural collapse and balancing the reaction evolution of metal oxides and organic ligands to facilitate ordered interfacial transformation.

As a typical ionic crystal, sodium chloride features low cost, easy removal by water washing and non-toxicity. With a melting point of 800 °C, it maintains stable structural protection within the calcination temperature range and inhibits particle agglomeration. Moreover, sodium chloride acts as an intercalation medium to induce porous structure formation during heat treatment. Accordingly, carbon nanomaterials with controllable morphology and pore structure can be selectively fabricated via salt recrystallization strategy.

Text S2 Analysis Method

Electron paramagnetic resonance (EPR) spectrometer was used to detect reactive oxygen species. For singlet oxygen detection, 30 μL reaction solution was mixed with 50 μL 100 mM TEMP trapping agent. The mixture was loaded into capillary tube, sealed with vacuum grease and placed in quartz tube for EPR measurement. For superoxide radical and alkoxyl radical detection, 30 μL reaction solution was blended with 30 μL 100 mM DMPO methanol solution, then sealed and tested in resonant cavity.

High performance liquid chromatography-mass spectrometry (HPLC-MS) was applied to identify degradation intermediates and deduce tetracycline degradation pathways. Test conditions: Waters BEH C18 column, injection volume 5 μL, column temperature 30 °C, flow rate 0.3 mL/min, analysis duration 15 min. Mobile phase consisted of 0.1% formic acid aqueous solution and acetonitrile. Mass spectrometry operated in electrospray positive ion mode with ionization voltage of 4000 V and mass scanning range of 50~1000 m/z.

Figure S1 The Schematic chart of HNC-T_x-Cl

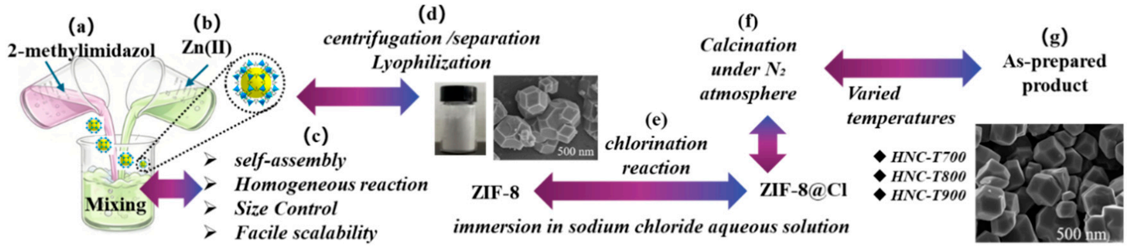

Figure S1 The Schematic chart of HNC-T_x-Cl

Figure S2 Full XPS Survey Spectrum of HNC-800-Cl after Tetracycline Degradation

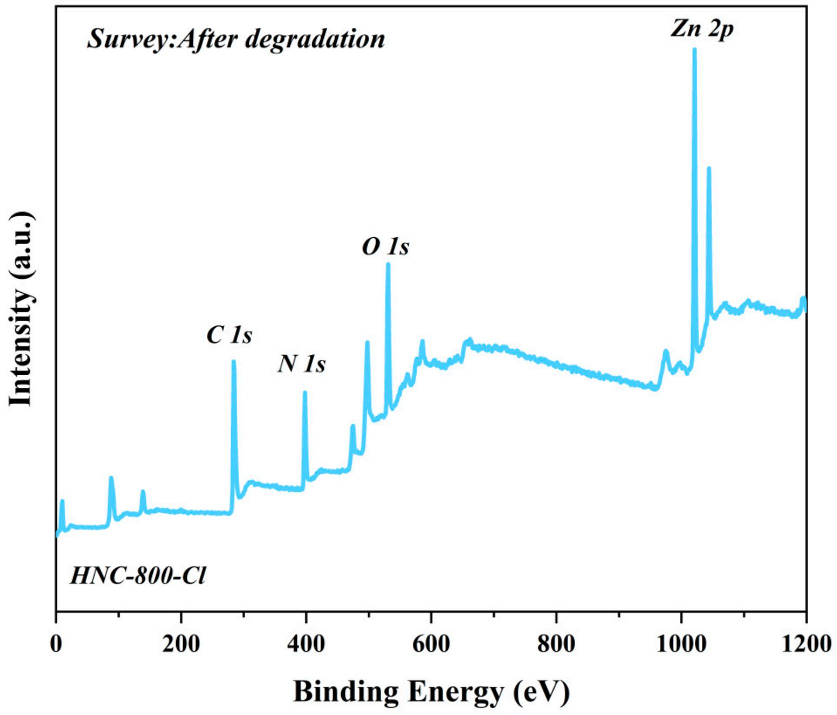

Figure S2 Full XPS Survey Spectrum of HNC-800-Cl after Tetracycline Degradation

Figure S3 High-resolution C 1s XPS spectrum of HNC-800-Cl after Tetracycline degradation

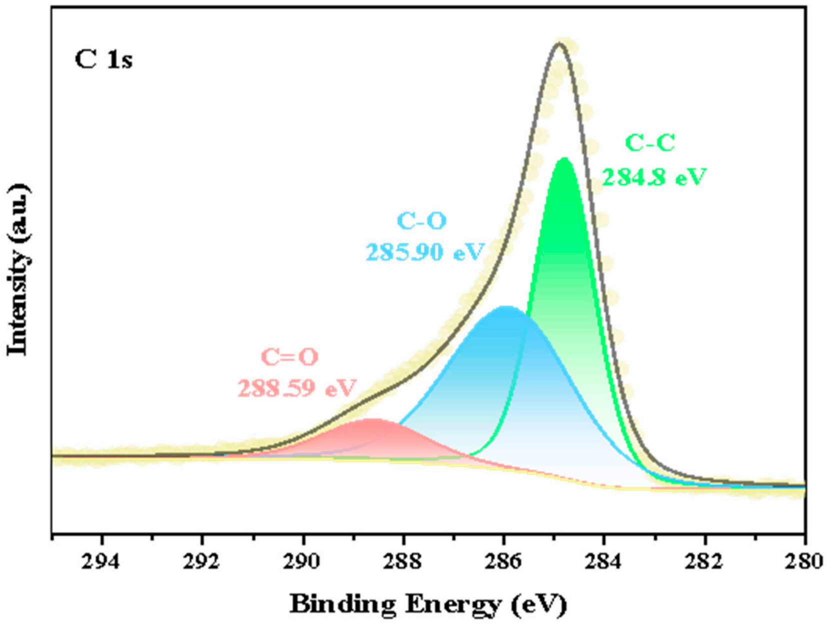

Figure S3 High-resolution C 1s XPS spectrum of HNC-800-Cl after Tetracycline degradation

Figure S4 High-resolution N 1s XPS spectrum of HNC-800-Cl after Tetracycline degradation

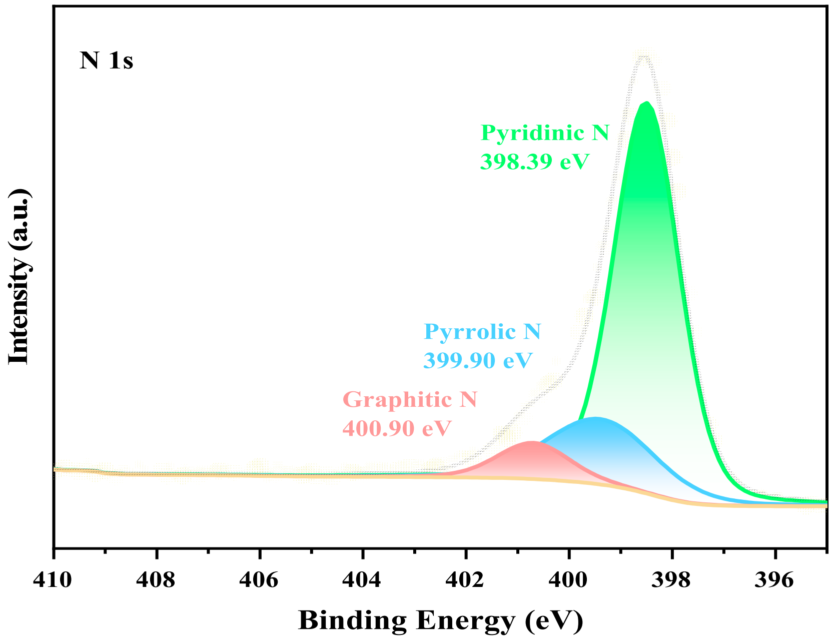

Figure S4 High-resolution N 1s XPS spectrum of HNC-800-Cl after Tetracycline degradation

Figure S5 High-resolution O 1s XPS spectrum of HNC-800-Cl after Tetracycline degradation

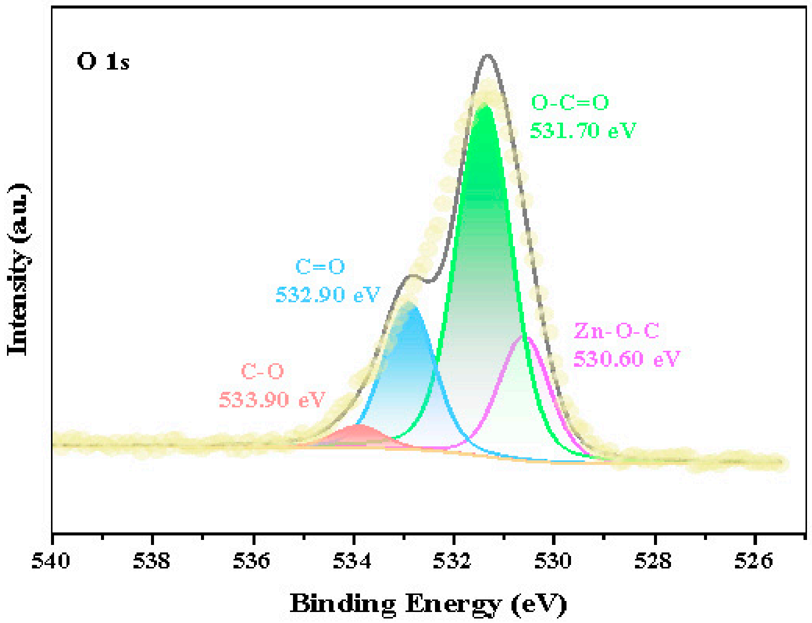

Figure S5 High-resolution O 1s XPS spectrum of HNC-800-Cl after Tetracycline degradation

Figure S6 Raman spectra of HNC-700-Cl and HNC-900-Cl

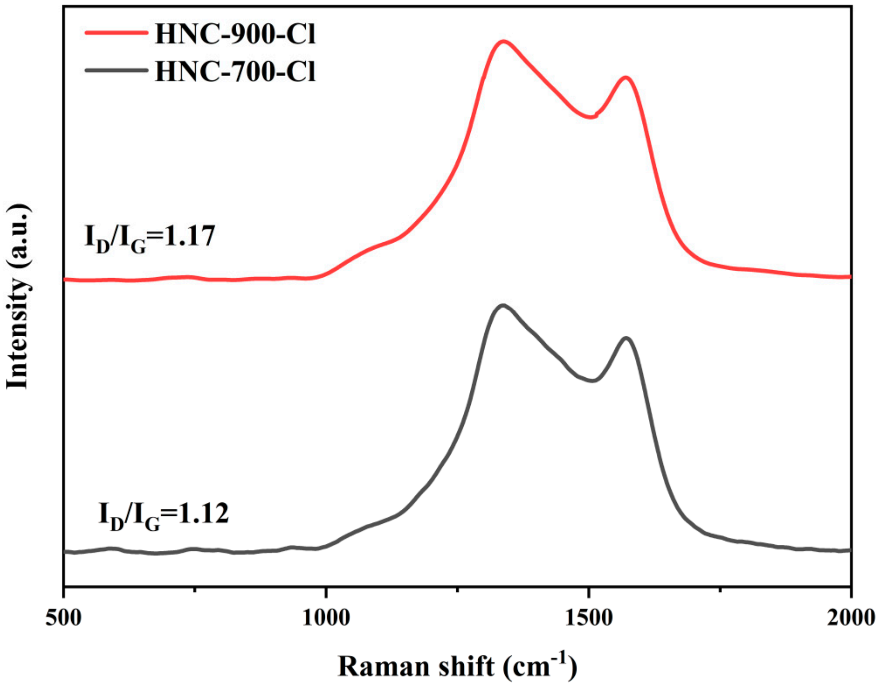

Figure S6 Raman spectra of HNC-700-Cl and HNC-900-Cl

Figure S7 N_2 adsorption-desorption isotherms of HNC-700-Cl and HNC-900-Cl

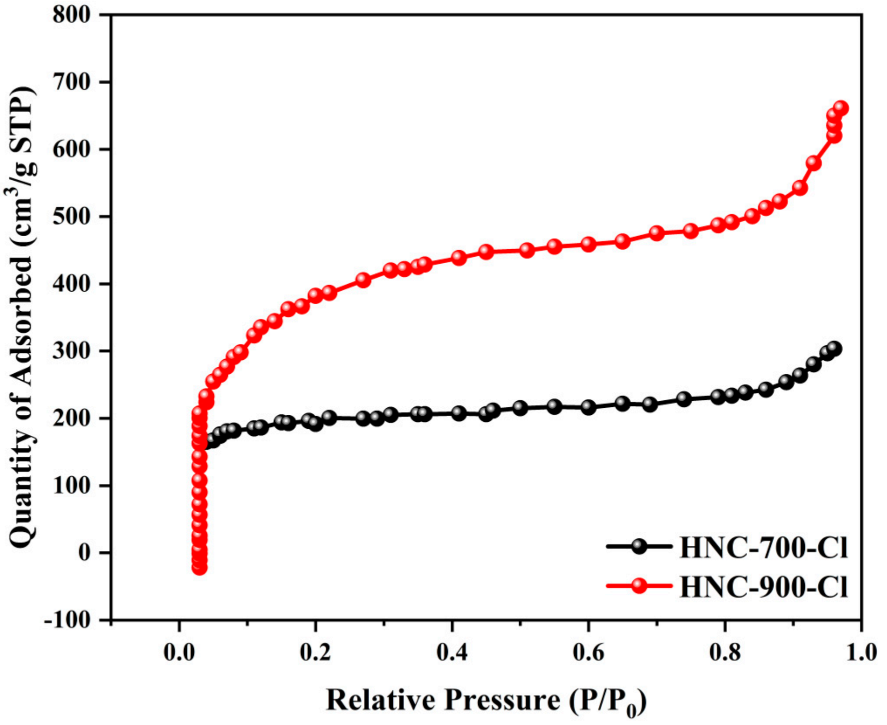

Figure S7 N_2 adsorption-desorption isotherms of HNC-700-Cl and HNC-900-Cl

Figure S8 Pore size distribution of HNC-700-Cl and HNC-900-Cl

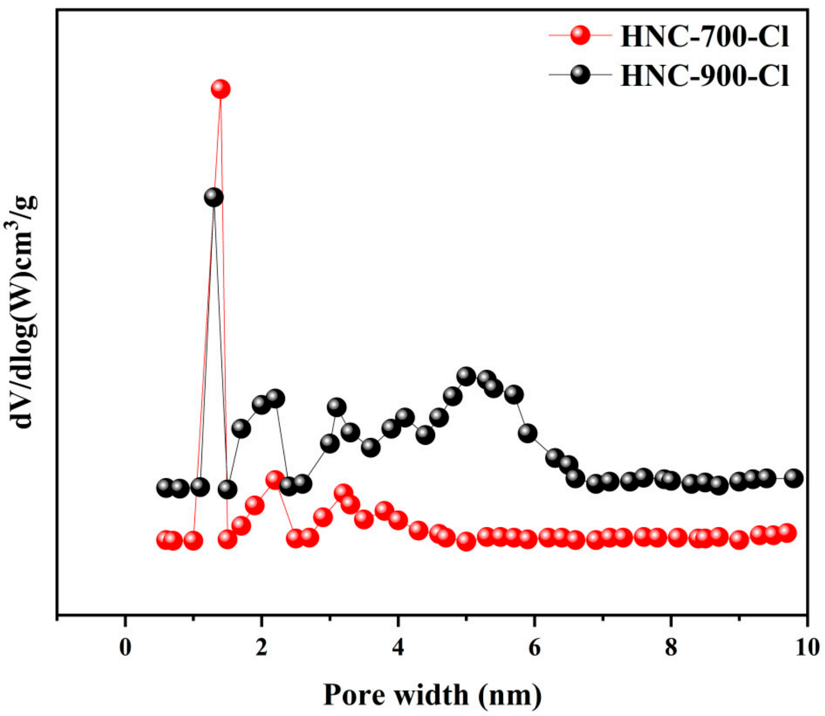

Figure S8 Pore size distribution of HNC-700-Cl and HNC-900-Cl

Figure S9 XRD pattern of ZIF-8

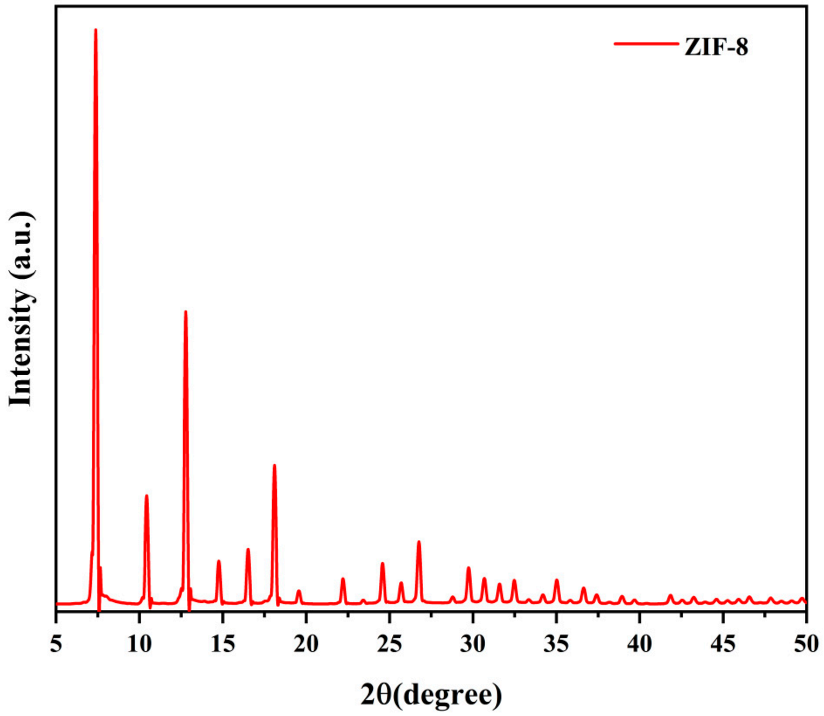

Figure S9 XRD pattern of ZIF-8

Figure S10 (a) The structures of TC and radicals;(b) The potential energy surface for the reaction between TC and radicals

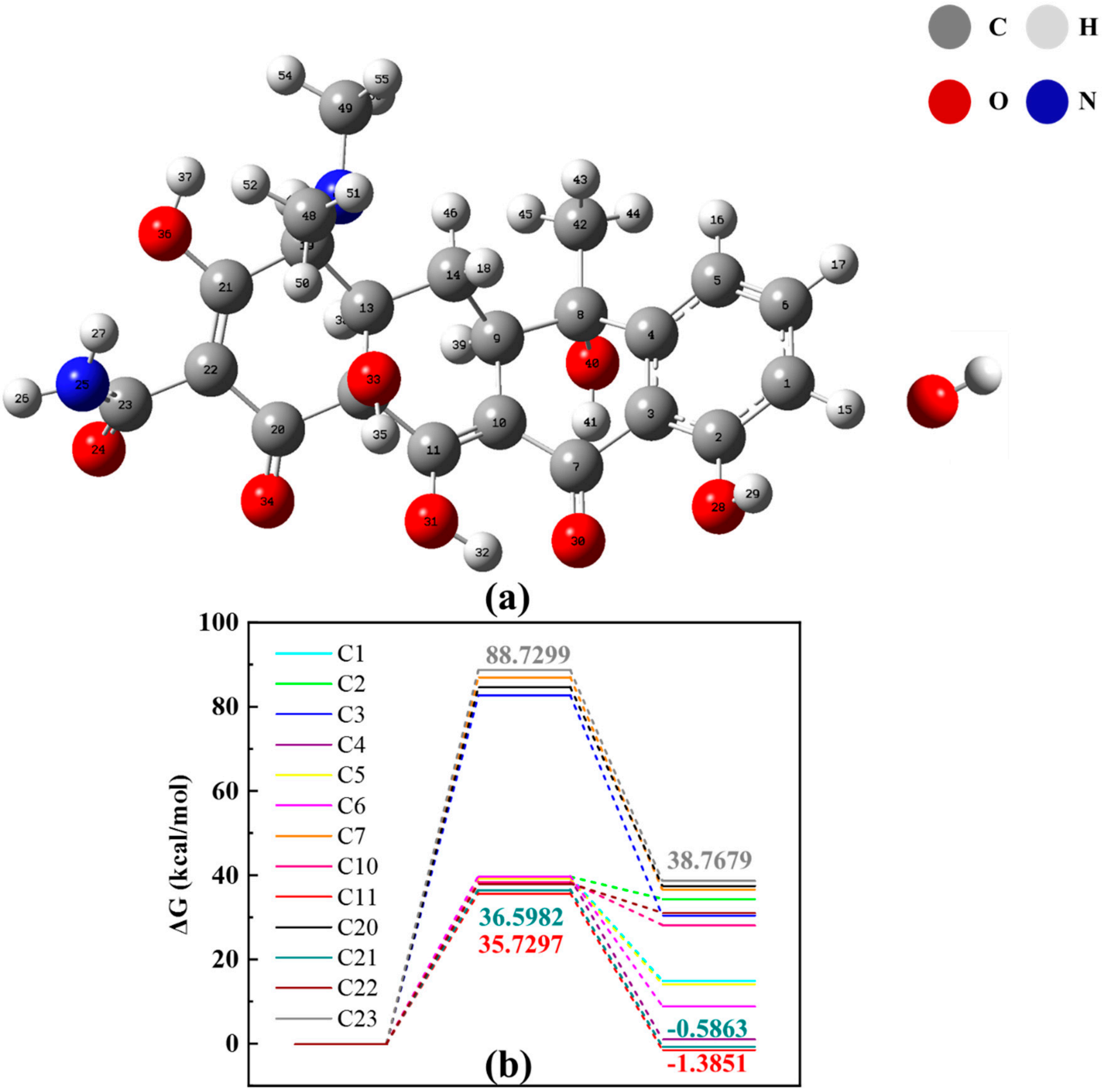

Figure S10 (a) The structures of TC and radicals;(b) The potential energy surface for the reaction between TC and radicals

Figure S11 Adsorption kinetic tests curve

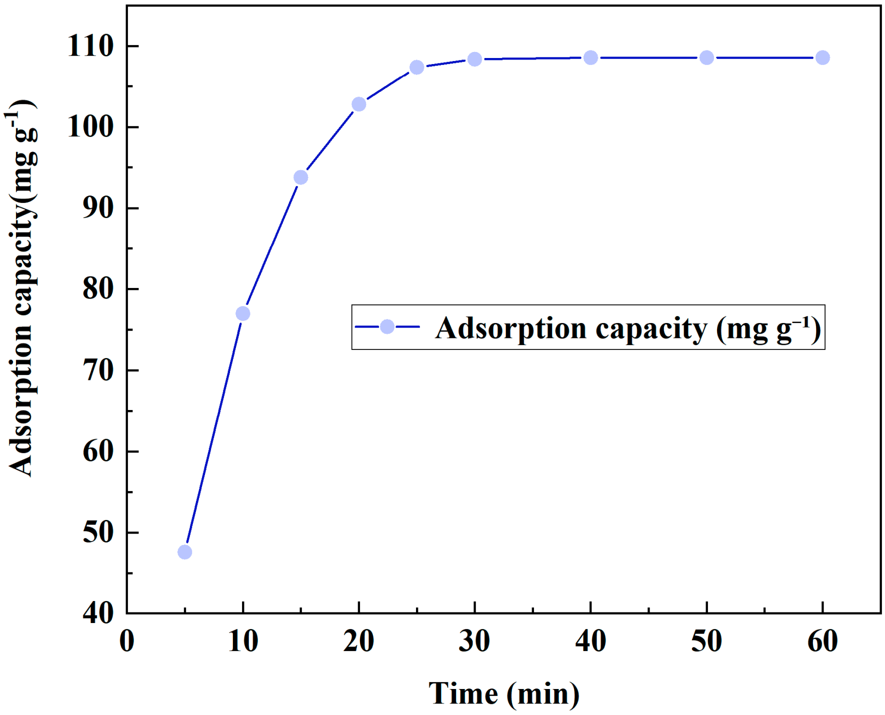

Figure S11 Adsorption kinetic tests curve

Table S1 Elemental proportion from XPS of NC and HNC-800-Cl

Table S1 Elemental proportion from XPS of NC and HNC-800-Cl

Samples	*C Content (%)*	*N Content(%)*	*O Content (%)*	*Zn Content (%)*
NC-800	73.12	12.6	9.89	4.39
HNC-800-Cl	50.34	19.05	17.02	13.59

Table S2 Relative content of various carbon species in C 1s XPS spectra of NC and HNC-800-Cl

Table S2 Relative content of various carbon species in C 1s XPS spectra of NC and HNC-800-Cl

Samples	*C-C* *At%*	*C-O* *At%*	*C=O* *At%*	*π-π** *At%*
NC-800	61.81	20.09	11.75	6.35
HNC-800-Cl	58.89	29.77	9.73	5.47

Table S3 Relative proportion of different nitrogen species in N 1s XPS spectra of NC and HNC-800-Cl

Table S3 Relative proportion of different nitrogen species in N 1s XPS spectra of NC and HNC-800-Cl

Samples	*Pyrodinic N* *At%*	*Pyrilic N* *At%*	*Grapgitic N* *At%*	*Oxidized N* *At%*
NC-800	68.33	12.55	9.70	9.42
HNC-800-Cl	74.25	15.12	7.72	2.91

Table S4 Relative content of various oxygen species in O 1s XPS spectra of NC and HNC-800-Cl

Table S4 Relative content of various oxygen species in O 1s XPS spectra of NC and HNC-800-Cl

Samples	*Zn-O-C*<br>*At%*	*O-C=O*<br>*At%*	*C=O*<br>*At%*	*C-O*<br>*At%*
NC-800	18.44	34.57	25.71	21.28
HNC-800-Cl	31.04	50.41	11.42	7.13

Table S5 Elemental composition of HNC-800-Cl after degradation via XPS analysis

Table S5 Elemental composition of HNC-800-Cl after degradation via XPS analysis

Samples	*C Content (%)*	*N Content (%)*	*O Content (%)*	*Zn Content (%)*
HNC-800-Cl	52.44	17.16	19.23	11.17

Table S6 Relative content of carbon species in C 1s XPS spectra of HNC-800-Cl after degradation

Table S6 Relative content of carbon species in C 1s XPS spectra of HNC-800-Cl after degradation

Samples	*C-C* *At%*	*C-O* *At%*	*C=O* *At%*	*π-π** *At%*
HNC-800-Cl	44.75	45.42	9.83	/

Table S7 Relative content of nitrogen species in N 1s XPS spectra of HNC-800-Cl after degradation

Table S7 Relative content of nitrogen species in N 1s XPS spectra of HNC-800-Cl after degradation

Samples	*Pyrodinic N*<br>*At%*	*Pyrilic N*<br>*At%*	*Grapgitic N*<br>*At%*	*Oxidized N*<br>*At%*
HNC-800-Cl	72.56	20.04	7.40	/

Table S8 Relative content of oxygen species in O 1s XPS spectra of HNC-800-Cl after degradation

Table S8 Relative content of oxygen species in O 1s XPS spectra of HNC-800-Cl after degradation

Samples	*Zn-O-C* *At%*	*O-C=O* *At%*	*C=O* *At%*	*C-O* *At%*
HNC-800-Cl	18.49	56.47	21.78	3.26

Table S9 Pore structure parameters of NC-800 and HNC-T_x-Cl

Table S9 Pore structure parameters of NC-800 and HNC-T_x-Cl

Catalyst	*S_{BET} (cm^2/g)*	*Pore volume (cm^3/g)*	*Pore diameter (nm)*
NC-800	976	0.32	6.2
HNC-700-Cl	892	0.41	6.7
HNC-800-Cl	1115	0.56	5.1
HNC-900-Cl	1348	1.01	4.4

Table S10 Main intermediates during tetracycline degradation in HNC-800-Cl/PMS system

Table S10 Main intermediates during tetracycline degradation in HNC-800-Cl/PMS system

Products	*m/z*	*Chemical formular*	*Chemical structure*
P1	445	$C_{23}H_{26}N_2O_8$	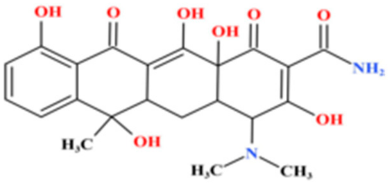
P2	416	$C_{20}H_{20}N_2O_8$	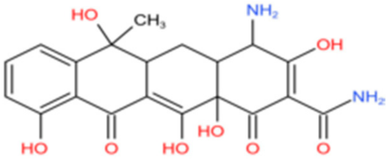
P3	362	$C_{19}H_{23}NO_6$	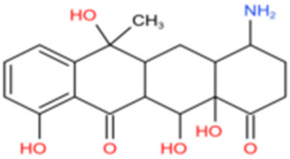
P4	282	$C_{15}H_{20}O_5$	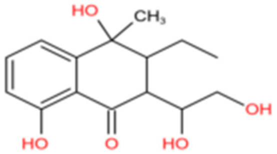
P5	340	$C_{19}H_{17}NO_5$	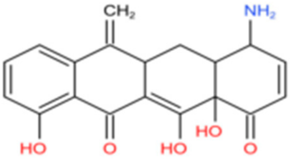
P6	184	$C_9H_{12}O_4$	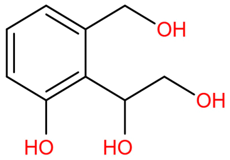
P7	105	$C_4H_8O_3$	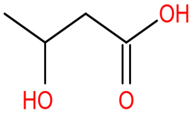
P8	414	$C_{21}H_{22}N_2O_7$	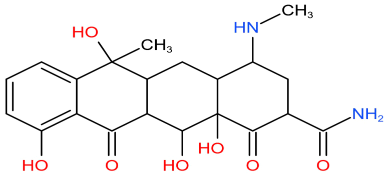

Table S10 Main intermediates during tetracycline degradation in HNC-800-Cl/PMS system(continue)

Products	*m/z*	*Chemical formular*	*Chemical structure*
P9	348	$C_{19}H_{24}O_6$	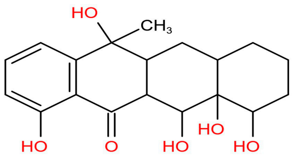
P10	308	$C_{17}H_{24}O_5$	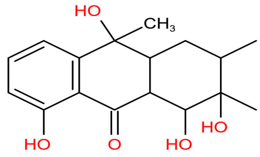
P11	262	$C_{15}H_{18}O_4$	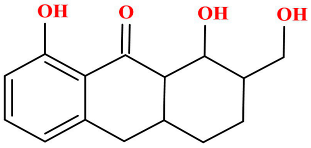
P12	162	$C_{10}H_{10}O_2$	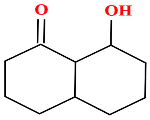
P13	460	$C_{22}H_{24}N_2O_9$	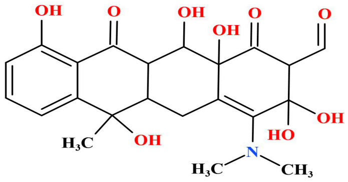
P14	491	$C_{22}H_{22}N_2O_{11}$	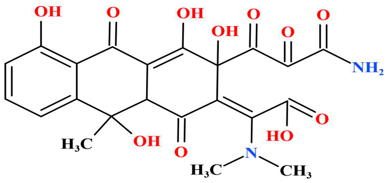
P15	477	$C_{21}H_{22}N_2O_9$	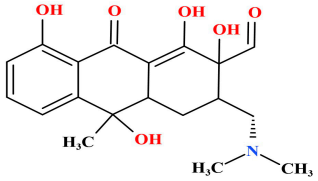
P16	389	$C_{19}H_{20}N_2O_7$	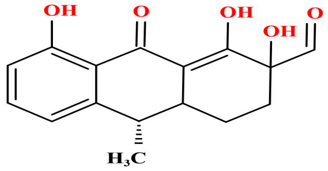

Table S10 Main intermediates during tetracycline degradation in HNC-800-Cl/PMS system(continue)

Products	*m/z*	*Chemical formular*	*Chemical structure*
P17	430	$C_{21}H_{22}N_2O_8$	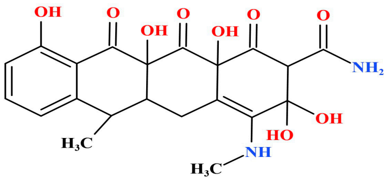
P18	350	$C_{20}H_{17}NO_6$	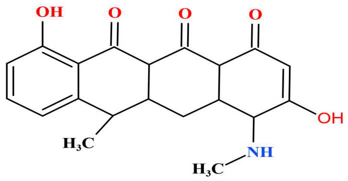

Table S11 Comparison of toxicity assessment[1,2]

Table S11 Comparison of toxicity assessment

Catalyst & Reaction System	*Key Toxicity Assessment Results*	*Environmental Safety Conclusion*	*Reference*
ZIF-8@NH₂-MIL-101(Fe) S-scheme MOF-on-MOF heterojunction, visible-light photo-Fenton system	The generated $\cdot O_2^-$ and $\cdot OH$ can efficiently destroy the conjugated tetracyclic skeleton of tetracycline hydrochloride; no significant biotoxicity was detected in the degradation intermediates	This photo-Fenton system can simultaneously achieve efficient degradation of tetracycline hydrochloride and toxicity reduction, with no obvious secondary pollution risk	[3]
Fluorine and nitrogen dual-doped metal-free carbon material (F,N-CM), peroxymonosulfate (PMS) activation system	The system degrades tetracycline via a non-radical pathway dominated by singlet oxygen ($^1O_2$) and electron transfer; no metal ion leaching occurs, and the toxicity of degradation products is significantly reduced	This metal-free catalytic system completely avoids the secondary pollution risk caused by metal leaching; the ecological toxicity of wastewater is greatly reduced after degradation, with excellent environmental safety	[4]
Hierarchical cobalt-based N-doped carbon catalyst (0.5Co-900), PMS activation system	1. Software prediction shows that the acute toxicity of tetracycline ring-opening intermediates to aquatic organisms is reduced by more than 2 orders of magnitude compared with the raw tetracycline; 2. Mung bean germination experiments confirm that the inhibitory effect of degraded wastewater on seed germination rate and root growth is almost completely eliminated	This system can efficiently degrade tetracycline and simultaneously achieve toxicity detoxification; the catalyst can be magnetically recovered with extremely low cobalt ion leaching, and has good engineering application safety	[5]
Chlorine-doped ZIF-8 derived carbon material (HNC-800-Cl), PMS activation system	The system degrades tetracycline via a radical + non-radical dual pathway dominated by superoxide radical ($\cdot O_2^-$); the tetracycline removal rate reaches 93.39% within 90 min, the conjugated toxic skeleton is completely destroyed, and no significant biotoxicity is detected in the degradation intermediates	This modified carbon material catalytic system can efficiently degrade tetracycline and reduce the ecological toxicity of wastewater, with no metal leaching risk and good environmental safety	This work

Table S12 The Zn leaching concentrations

Table S12 The Zn leaching concentrations

pH	*Residual Zn^{2+} concentration (mg L^{-1})*
3	0.21
5	0.13
7	0.06
9	0.09

Table S13 Comparison of Cl-doped ZIF-8 derived carbon (HNC-800-Cl) and other materials

Table S13 Comparison of Cl-doped ZIF-8 derived carbon (HNC-800-Cl) and other materials

Catalytic Material	*Activator/System*	*Main Reaction Conditions*	*TC Removal Efficiency (90 min)*	*Reference*
Cl-doped ZIF-8 derived carbon (HNC-800-Cl)	PMS	Catalyst=0.05 g/L, PMS=0.1 mM/L, pH=7	93.39%	This work
High-redox ZIF-8@NH_2-MIL-101(Fe) S-scheme MOF-on-MOF heterojunction	Visible light photocatalysis	Catalyst=0.10 g/L, λ≥420 nm visible light, pH=7	91.40%	[3]
F/N dual-doped ZIF-derived carbon	PMS	Catalyst=0.10 g/L, PMS=0.4 mM/L, pH=7	92.00%	[4]
Hierarchical Co-based N-doped carbon from hybrid MOF	PMS	Catalyst=0.10 g/L, PMS=0.4mM, pH=7	96.10%	[5]

Reference:

1. Wei, L.; Liu, S.; Nguyen, V.; Zheng, M.; Wang, H. Visible-light driven O2-to-H2O2 synchronized activation of peroxymonosulfate in Z-scheme photocatalytic fuel cell for wastewater purification with power generation. *Applied Catalysis B-Environment and Energy* 2025, 361,124594, http://dx.doi.org/10.1016/j.apcatb.2024.124594.
2. Wang, X.; Luo, X.; Li, R.; Chang, Y.; Peng, J.; Wang, W.; Liu, H.; Yan, G.; Wei, P.; Cao, Z. Boosting peroxymonosulfate activation over partial Zn-substituted Co3O4 for florfenicol degradation: Insights into catalytic performance, degradation mechanism and routes. *Chemical Engineering Journal* 2024, 491, 152197, http://dx.doi.org/10.1016/j.cej.2024.152197.
3. Wang, Y.; Lu, C.; Jiao, D.; Wang, F.; Gong, X.; Yu, T.; Wang, A.; Wang, C. Efficient degradation of tetracycline hydrochloride via high-redox S-scheme MOF-on-MOF heterojunction catalyst. *Chinese Chemical Letters* 2026, 37, 111979, http://dx.doi.org/10.1016/j.cclet.2025.111979.
4. Huang, L.; Gong, X.; Xie, J.; Zhang, L.; Luo, X. Fluorine and nitrogen dual-doped carbon material as metal-free peroxymonosulfate activator for efficient tetracycline degradation: Radical-free mechanism. *Chemical Engineering Science* 2023, 280, 118979, http://dx.doi.org/10.1016/j.ces.2023.118979.
5. Cao, X.; Liu, Y.; Feng, T.; Huang, X.; Qiu, W. Enhancing peroxymonosulfate activation with hierarchical cobalt-based N-doped carbon catalysts derived from hybrid MOFs intermediates for tetracycline degradation: Performance, mechanism insights and biotoxicity evaluation. *Journal of Water Process Engineering* 2025, 71, 107383, http://dx.doi.org/10.1016/j.jwpe.2025.107383.
